# Factors driving adaptive radiation in plants of oceanic islands: a case study from the Juan Fernández Archipelago

**DOI:** 10.1007/s10265-018-1023-z

**Published:** 2018-03-13

**Authors:** Koji Takayama, Daniel J. Crawford, Patricio López-Sepúlveda, Josef Greimler, Tod F. Stuessy

**Affiliations:** 1 Graduate School of Science, Kyoto University, Kitashirakawa Oiwake-cho, Sakyo-ku, Kyoto, 606-8502 Japan; 2 Department of Ecology and Evolutionary Biology, Biodiversity Institute, University of Kansas, Lawrence, KS 60045 USA; 3 Department of Botany, University of Concepción, Casilla 160-C, Concepción, Chile; 4 Department of Botany and Biodiversity Research, University of Vienna, Rennweg 14, 1030 Vienna, Austria; 5 Herbarium and Department of Evolution, Ecology, and Organismal Biology, The Ohio State University, 1315 Kinnear Road, Columbus, OH 43212 USA

**Keywords:** Adaptation, Anagenesis, Biogeography, Cladogenesis, Robinson Crusoe Islands, Speciation

## Abstract

Adaptive radiation is a common evolutionary phenomenon in oceanic islands. From one successful immigrant population, dispersal into different island environments and directional selection can rapidly yield a series of morphologically distinct species, each adapted to its own particular environment. Not all island immigrants, however, follow this evolutionary pathway. Others successfully arrive and establish viable populations, but they remain in the same ecological zone and only slowly diverge over millions of years. This transformational speciation, or anagenesis, is also common in oceanic archipelagos. The critical question is why do some groups radiate adaptively and others not? The Juan Fernández Islands contain 105 endemic taxa of angiosperms, 49% of which have originated by adaptive radiation (cladogenesis) and 51% by anagenesis, hence providing an opportunity to examine characteristics of taxa that have undergone both types of speciation in the same general island environment. Life form, dispersal mode, and total number of species in progenitors (genera) of endemic angiosperms in the archipelago were investigated from literature sources and compared with modes of speciation (cladogenesis vs. anagenesis). It is suggested that immigrants tending to undergo adaptive radiation are herbaceous perennial herbs, with leaky self-incompatible breeding systems, good intra-island dispersal capabilities, and flexible structural and physiological systems. Perhaps more importantly, the progenitors of adaptively radiated groups in islands are those that have already been successful in adaptations to different environments in source areas, and which have also undergone eco-geographic speciation. Evolutionary success via adaptive radiation in oceanic islands, therefore, is less a novel feature of island lineages but rather a continuation of tendency for successful adaptive speciation in lineages of continental source regions.

## Introduction

Oceanic islands have long been regarded as suitable locations to examine processes of organic evolution (Losos and Ricklefs 2009; Wallace 1881). Studies in the twentieth century have focused on understanding spectacular examples of evolution such as the silverswords, lobelioids, and *Drosophila* in the Hawaiian Islands (Carlquist et al. 2003; Carson 1982; Givnish et al. 2009), Darwin’s finches in the Galápagos Islands (Grant 1986, 1998; Grant and Grant 2008, 2014), and *Aeonium* in the Canary Islands (Jorgensen and Olesen 2001). Increased precision in assessing relationships has now been made possible by employment of a variety of molecular markers (Baldwin et al. 1998; Emerson 2002; Mort et al. 2015; Ronquist and Sanmartin 2011).

There are two important steps for understanding island biota. The first step has often been to describe the relationships among island endemics using phylogenetic analysis (Glor 2010). An important concern is to ascertain whether the group under consideration is monophyletic, which would be suggestive of a single origin to the archipelago (e.g., in the silverswords of the Hawaiian Islands, Baldwin 2003; *Sonchus* of the Canary Islands; Kim et al. 1996a, b, 1999; *Dendroseris* and *Robinsonia* of the Juan Fernández Islands; Sang et al. 1994, 1995; more examples in; Baldwin et al. 1998). The second step is to attempt to understand processes of evolution, especially speciation. Molecular markers now provide avenues for more precisely evaluating genetic variation within and among populations (Avise 2004; Freeland et al. 2011; Żukowska and Wachowiak 2016), which facilitates inferring modes of speciation. From a general perspective, two patterns of speciation can be identified: cladogenesis and anagenesis (Stuessy et al. 1990). The former exists when an immigrant lineage splits into two or more populational systems that eventually diverge into distinct species. If this process is rapid and accompanied by adaptation to diverse habitats, and with conspicuous morphological change, it is referred to as adaptive radiation (Gavrilets and Losos 2009; Schluter 2000). This is the pattern of speciation so typical of the dramatic examples of island evolution mentioned above. Another major process of speciation is anagenesis (Stuessy et al. 2006), or transformational speciation, in which an island immigrant lineage does not split and diverge, but remains as a single large population and slowly accumulates genetic variation via mutation and recombination shaped by drift and/or selection. The end result from this process is a single new species as measured by distinct morphological and genetic characters in comparison with continental progenitors. This is one type of progenitor-derivative speciation (Crawford 2010).

The existence of two major processes of speciation in plants of oceanic archipelagos raises the question of why some immigrant populations diverge dramatically via adaptive radiation and others do not. The island environment clearly plays an important role. In comparison of islands that are environmentally heterogeneous with those that are homogeneous, the former provides opportunities for adaptive radiation and the latter does not (Givnish 2010; Rainey and Travisano 1998; Steinbauer et al. 2016; Stuessy et al. 2006). A good example of an island environment in which cladogenesis and adaptive radiation are virtually unknown is Ullung Island, a low and young island (Kim 1985a, b) off the coast of Korea and relatively ecologically uniform (Kim 1988; Stuessy et al. 2006; Takayama et al. 2016). In contrast, in islands that have a rich diversity of environments, such as in the Hawaiian or Canarian Archipelagos, abundant cladogenesis and adaptive radiation have occurred (Stuessy et al. 2006). Continuing natural disturbances such as hurricanes, volcanic activity, and landslides have also taken place in these islands, which have created open habitats that may have stimulated genetic heterogeneity and morphological divergence (Robichaux et al. 1990; Whittaker 1995).

There are many archipelagos, however, in which both cladogenesis and anagenesis have taken place (Stuessy et al. 2006). These might be favorable locations in which to identify the factors that drive adaptive radiation because the island environments are available for all immigrants, although the existing ecology will be modified over geological time. A comparison of factors in groups that have adaptively radiated with those that have only undergone anagenesis in the same archipelago might offer clues to the reasons for adaptive radiation or lack thereof. Biological attributes of the immigrants, as well as characteristics of progenitors, plus ability to disperse into and adapt to diverse island habitats, would all likely be relevant.

**Fig. 1:**
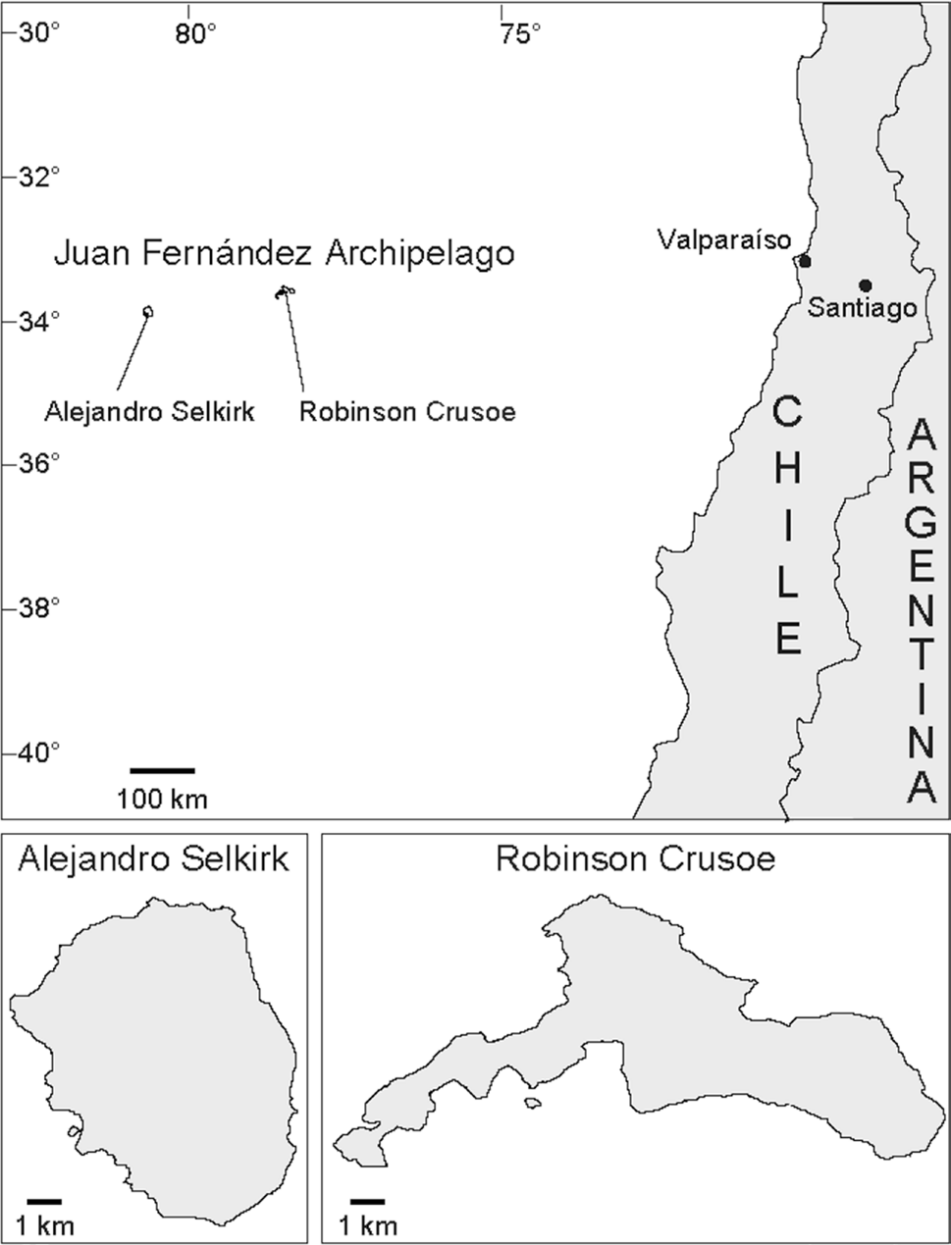
Location of the Juan Fernández Archipelago in the southeastern Pacific Ocean

A suitable archipelago in which to seek factors responsible for promoting adaptive radiation is the Juan Fernández (= Robinson Crusoe) Archipelago (Fig. 1) (Stuessy et al. 2017a). Situated 687 kms west of continental Chile at 33°S latitude, the archipelago consists of two main islands: Robinson Crusoe (= Masatierra) and Alejandro Selkirk (= Masafuera) Island. Although these are now approximately of equivalent size (c. 50 km^2^ each; Stuessy 1995), they are of different geological ages, with the former 4 million years old and the latter 1–2 million (Stuessy et al. 1984). From a biogeographic perspective, this is a favorable setting, as the island closer to the continent from where the majority of the immigrants have come (Bernardello et al. 2006) is also the oldest. It is also estimated to have been much larger at its geological origin (Stuessy et al. 1998). The probability of an immigrant arriving first to Robinson Crusoe Island, therefore, is vastly greater than to Alejandro Selkirk Island.

Considerable comparative phylogenetic research has already been accomplished on endemic plants of the Juan Fernández Archipelago (e.g., Crawford et al. 1998; López-Sepúlveda et al. 2014; Ruiz et al. 2004; Stuessy et al. 2005; Takayama et al. 2014). This allows comparisons to be made with direct ancestors in the mainland, especially regarding biological attributes. It also identifies sister species in the islands, so important for evaluating modes of speciation. Furthermore, assessments have been made on which generic groups have evolved via cladogenesis or anagenesis (Stuessy et al. 1990), which provides a structure of categories by which to make direct comparisons. The big advantage is that within the endemic flora of these islands, both cladogenesis and anagenesis have been responsible for providing the specific diversity that is seen today.

A number of evolutionarily relevant studies already have been conducted on endemic species of the Juan Fernández Islands. Detailed population genetic investigations have been carried out with isozymes, AFLPs and microsatellites (Crawford et al. 2001; López-Sepúlveda et al. 2014; Takayama et al. 2014). These studies have allowed a better understanding of patterns of genetic variation over the island landscapes, which is important for understanding speciation. Furthermore, chromosomal studies (Kiehn et al. 2005; Sanders et al. 1983; Spooner et al. 1987; Sun et al. 1990) have surveyed the endemic flora to reveal possible mechanisms of speciation due to chromosomal change. Secondary product chemistry (flavonoids) has also been surveyed in several endemic genera at the populational level (e.g., Pacheco et al. 1985, 1991; Ruiz et al. 1994). Lastly, modern vegetation analyses have been done on both islands (Greimler et al. 2002, 2013), which provide a uniform measure of ecological zonation over both islands. This is fundamental for addressing issues associated with adaptive radiation.

The objectives of this present paper, therefore, are to: (1) outline the genera in the Juan Fernández Archipelago in which anagenetic and cladogenetic speciation (and adaptive radiation) have occurred; (2) examine the biological features of these taxa to understand better their potential for undergoing different types of speciation, especially cladogenesis; (3) investigate the ecological context for each of the generic groups that have adaptively radiated in the islands; and (4) suggest the factors that appear to be significant for driving adaptive radiation in these island plant taxa.

## Endemic angiosperms in the Juan Fernández Islands

To address aspects of speciation and adaptive radiation in the flora of any oceanic island, a categorization of all endemic species must first be accomplished. Deciding whether a species has had a cladogenetic or anagenetic origin is superficially simple (in general, one vs. two or more endemic species), but some cases can be difficult. For categorization in the Juan Fernández Islands, if a single endemic species occurs on one or both islands, we assume an anagenetic origin. One might argue that two endemic species in the archipelago, one on each of the islands, might indicate a cladogenetic event, especially if both islands were colonized at the same time and diverged in isolation. That Robinson Crusoe Island is older and nearer the continent, however, makes this much less probable. In any event, the process of speciation on separate islands is still anagenetic. If two or more species exist on a single island, we accept they have originated cladogenetically. They must, obviously, have originated from a single original introduction. Two species would not usually be regarded as an example of adaptive radiation, as normally three or more species are involved, but certainly two species on an island reflect cladogenesis. Phylogenetic studies on the endemic flora of the Juan Fernández Islands (see reviews in Ruiz et al. 2004; Stuessy et al. 2005, 2017b) allow greater precision with the endemic flora. For example, *Dendroseris* has three species on the younger island, but we know from several phylogenetic studies (Crawford et al. 1998; Sanders et al. 1987; Sang et al. 1994) that these are each anagenetically derived from different subgenera on the older island. We also know, for example, that in the case of *Myrceugenia*, with one species on each island, that these are not at all closely related to each other (Murillo-Aldana et al. 2012, 2013). *Myrceugenia fernandeziana*, in fact, has been suggested to belong in a different genus (placed in *Nothomyrcia* by Murillo-Aldana and Ruiz 2011).

These cases emphasize the importance of using all available data regarding phylogenetic relationships and populational genetic diversity to best assess modes of speciation in these island floras.

This simple and direct approach to categorization of patterns of speciation does not mean that more complex scenarios could not have occurred. Single species could be only surviving lineages of much larger adaptively radiated complexes in the past. Two (or more) species on the same island, if poorly understood phylogenetically, could reflect independent cases of anagenesis. It is also possible that a group in an island could diversify by cladogenesis but not adaptively radiate in the process. Although all these scenarios are possible, the normally diverse habitats present in oceanic islands, especially in those with elevations over 1000 m or with deep and separated canyons, provide opportunities for adaptation of lineages to available ecological zones. In the face of no evidence to the contrary, we believe that the approach used here is reasonable and does allow for valid inferences to be made about the factors involved with adaptive radiation, which is the focus of this paper.

Tables 1 and 2 provide lists of all the genera of angiosperms that have produced endemics in the Juan Fernández Islands. Those that have anagenetic origins are given in Table 1, and those that have originated cladogenetically, and adaptively radiated, are given in Table 2. Data show that 51 taxa (c. 49%) have originated cladogenetically and 52 taxa (c. 51%) anagenetically (Stuessy et al. 2017b: table 13.7). That the endemic flora separates well into two major elements provides a good database for comparison between the two processes. These data also emphasize the importance of anagenesis as a mode of speciation in the archipelago.

**Table 1:** Genera of angiosperms in the Juan Fernández Archipelago that have speciated anagenetically

Genera	Life form typical of genus	Total number of species in progenitor genus	Dispersal mode
One species on Robinson Crusoe Island			
*Azara* (var.)	Herbs	10 spp	Endozoochory
*Boehmeria*	Herbs to trees	80 spp	Anemochory
*Chusquea*	Cane (bamboos)	137 spp	Anemochory (endozoochory)
*Colletia*	Trees	5 spp	Autochory
*Dysopsis*	Herbs	3 spp	Autochory
*Escallonia*	Shrubs & trees	39 spp	Anemochory
*Greigia*	Herbs	28 spp	Autochory
*Juania*	Palm	Endemic, in *Ceroxylon* group	Autochory
*Lactoris*	Subshrub	Endemic, in Piperales	Anemochory
*Machaerina*	Herbs	45 spp	Anemochory (endozoochory)
*Ochagavia*	Herbs	4 spp	Autochory
*Plantago*	Herbs, some rosette trees	270 spp	Anemochory
*Podophorus*	Herb	Endemic, extinct, possibly	Anemochory (endozoochory)
		related to *Megalachne*	
*Santalum*	Trees	25 spp., extinct in archipelago	Endozoochory
*Selkirkia*	Tree	Endemic, affinities unclear	Epizoochory
*Solanum*	Trees, shrubs, vines, herbs	1250 spp	Endozoochory
*Ugni*	Shrubs	4 spp	Endozoochory
One species on Alejandro Selkirk Island			
*Acaena*	Herbs	110 spp	Epizoochory
*Agrostis*	Herb	175 spp	Anemochory (endozoochory)
*Cardamine*	Herbs	200 spp	Autochory
*Empetrum*	Creeping shrubs	2 spp	Endozoochory
*Euphrasia*	Herbs	350 spp	Anemochory
*Gavilea*	Herbs	13 spp	Anemochory
*Luzula*	Herbs	108 spp	Anemochory
*Ranunculus*	Herbs	600 spp	Autochory
One (same) species on both islands			
*Carex*	Herbs	1800 spp	Anemochory (endozoochory)
*Drimys*	Trees	6 spp	Autochory
*Pernettya*	Shrubs	14 spp	Endozoochory
*Rhaphithamus*	Trees	2 spp	Autochory
Two different species, one on each island			
*Berberis*	Shrubs	600 spp	Endozoochory
*Haloragis*	Shrubs	28 spp	Autochory
*Myrceugenia*	Trees	40 spp	Endozoochory
*Nicotiana* (vars.)	Herbs & shrubs	76 spp	Anemochory
*Spergularia*	Herbs	60 spp	Autochory
*Zanthoxylum*	Shrubs & trees	200 spp	Endzoochory

Anagenesis is certified when there is not more than one species on one island. Inferences on mode of speciation from Stuessy et al. (2017b). The number of total species and life form in each genus is provided from Mabberley (2008) and other sources, dispersal mode from Bernardello et al. (2006)

**Table 2:** Genera of angiosperms in the Juan Fernández Archipelago that have speciated cladogenetically (involving adaptive radiation) or both cladogenetically and anagenetically, with numbers of species occurring on each island

Mode of speciation	RC Island	AS Island	Life form typical of genus	Total number of species in progenitor genus	Dispersal mode
Cladogenesis					
*Centaurodendron, Yunquea*	3		Herbs	Endemic, out of *Centaurea*, 650 spp	Autochory
*Chenopodium*	3		Herbs	100 spp	Anemochory
*Coprosma*	2		Herbs, shrubs, trees	100 spp	Autochory
*Cuminia*	2		Shrubs	Endemic, near *Monardella* and *Mintho-stachys*; 52 spp	Autochory
*Erigeron*	*	6	Herbs	390 spp	Anemochory
*Eryngium*	3		Herbs	250 spp	Epizoochory
*Urtica*	**	2	Mostly herbs	70 spp	Anemochory
Cladogenesis and anagenesis					
*Carex*	2	1	Herbs	2000 spp	Anemochory (endozoochory)
*Dendroseris*	8	3	Mostly herbs	Endemic, out of *Sonchus*, 62 spp	Autochory
*Gunnera*	2	1	Herbs	40 spp	Autochory
*Megalachne*	2	1	Herbs	Endemic, out of *Bromus* or *Festuca*, 150 or 450 spp	Anemochory (endozoochory)
*Peperomia*	3	1	Herbs	1600 spp	Epizoochory
*Robinsonia*	7	1	Herbs	Endemic, from *Senecio*; 1000 spp	Autochory
*Wahlenbergia*	3	2	Herbs and shrubs	125 spp	Anemochory

*Erigeron fernandezia also occurs on Robinson Crusoe Island, perhaps an historical introduction (López-Sepúlveda et al. 2015)

**Urtica glomerosa occurs also on Robinson Crusoe Island, but the reasons for this distribution are unknown

Cladogenesis is certified when there are two or more species within the same island. Inferences on type of species from Stuessy et al. (2017a). Species numbers from Mabberley (2008) and other sources, dispersal mode from Bernardello et al. (2006)

Several different patterns can be seen from the data in Tables 1 and 2. With anagenesis, many more genera have arrived on the closer and older island and anagenetically changed, yielding transformed species. Only eight genera have arrived preferentially to the younger island where they speciated anagenetically. As this island is both younger and also more distant from the principal source region in continental southern South America (Bernadello et al. 2006), this seems unsurprising. Furthermore, it has been hypothesized (Stuessy et al. 1998) that Robinson Crusoe Island has lost 95% of its surface area over its ontogeny through 4 million years, which means it was a much larger target area originally.

Only four genera exist in the islands with the same endemic species on both islands, *Carex, Drimys, Pernettya*, and *Rhaphithamnus*. Population genetic studies on *Drimys confertifolia* (López-Sepúlveda et al. 2014) reveal that the populations on each island do differ in genetic diversity, equivalent, in fact, to specific differences in other genera, such as *Erigeron* (López-Sepúlveda et al. 2015). No morphological differences, however, have been found between the two sets of populations, but genetic differentiation is clearly taking place in isolation (Stuessy and Crawford 2016). It would not be surprising if this were also the case with the other two genera. In fact, in *Rhaphithamnus*, divergence in neutral genetic markers is greater between populations in the two islands than between either and the continental sister species (López-Sepúlveda et al. 2016). Despite the divergence between the islands, no morphological differences have been detected. There are, however, morphological characters distinguishing the island species from their continental relative (Sun et al. 1996). It should be mentioned that three of these four genera have fleshy fruits, perhaps attractive to birds that could have flown to the younger island when it originated 1–2 million years ago.

Divergence to the specific level between islands has occurred in the cases of seven genera, i.e., *Berberis, Haloragis, Myrceugenia, Nicotiana* (vars.), *Sophora, Spergularia*, and *Zanthoxylum*. With *Myrceugenia*, it has already been mentioned that each species appears to have had an independent origin from the mainland (Murillo-Aldana et al. 2013). Detailed phylogenetic analyses of the species of *Sophora* and *Haloragis* have not yet been done to know what the specific origins might have been. *Haloragis* is one of the genera that has its origins from the western Pacific, as the genus is not known from South America. Depending upon when it immigrated to the archipelago, it could have arrived first on the younger island and then dispersed to the older island. The degree of morphological divergence between the two endemic species, however, suggests that the original immigrant probably first colonized Robinson Crusoe Island and then dispersed to and diverged on the younger island when it became available.

Fourteen genera have speciated cladogenetically in the Juan Fernández Islands (Table 2), most of them, except *Erigeron, Urtica*, and *Wahlenbergia*, on the older island (Robinson Crusoe). *Erigeron* has radiated on Alejandro Selkirk Island, which strongly suggests that it arrived there directly, rather than first immigrating to the older island and then traveling further westward to the younger island. Population genetic studies (López-Sepúlveda et al. 2015) revealed that molecular divergence among some of these species is not great, especially among those of the *E. ingae* complex (involving *E. ingae, E. turricola*, and *E. luteoviridis*), which is less than seen between island congeners in other genera. This is the only example of cladogenesis and adaptive radiation occurring on the younger island. Seven genera have speciated cladogenetically on Robinson Crusoe Island and also yielded anagenetically derived species on Alejandro Selkirk Island. These genera figure among the group that shows the highest cladogenetic speciation on this island, and that shows most evidence of adaptive radiation. For example, *Dendroseris* has three species on the younger island, but molecular phylogenetic studies (Sang et al. 1994) have shown that each species has been derived anagenetically from a different subgenus on the older island. *Wahlenbergia* has two endemic species on Robinson Crusoe Island and another two on Alejandro Selkirk Island, and the other three genera all have single species of anagenetic origin from the older island. A clear case is in *Robinsonia* whereby *R. masafuerae* has been derived from *R. evenia* on Robinson Crusoe Island (Sang et al. 1995; Takayama et al. 2014).

## Biological attributes of island endemics and their progenitors

Fundamental for understanding adaptive radiation in oceanic islands is to examine the biological features of groups that have successfully immigrated and radiated. Important in this regard are aspects of dispersal, habit, breeding systems, and pollination systems. Also significant are tendencies embedded in the phylogenetic history of the group, particularly those for allopatric homoploid speciation and ecological differentiation in parental populations. It is assumed that for adaptive radiation to take place, the island environment must be heterogeneous (Losos and Mahler 2010; Stuessy et al. 2006), with different habitats selecting for different morphological (or other) features (Givnish 2015; Soulebeau et al. 2015). It should be pointed out that in nearly all cases of adaptive radiation in any archipelago, we do not have real evidence of adaptations. That is, we see correlations of features with specific environments, but few experiments have been done to test fitness of these features in present and subsequent generations. For one good example in the Hawaiian lobelioids, however, see Givnish et al. (2004).

### General features of adaptively radiated plant groups

All endemic plants of oceanic islands must have been good dispersers, or they would not have successfully immigrated to the isolated islands. Some mechanisms of long-distance dispersal for seeds or fruits are obvious, such as attachment to bird feathers, or parasols for air flotation, but in some cases it is difficult to discern the mode of travel (Vargas et al. 2012). Once established in an island, however, the mechanisms of original dispersal may not be as useful for travel within (or among) islands. Carlquist (1974) has shown that many endemic species of oceanic archipelagos have probably been dispersed by birds. This would mean either fleshy fruits ingested or dried fruits attached to feet or feathers (more likely). In any event, if such a transport occurred with a bird blown off course by chance, this same means of transport would not usually be available for further intra- or inter-island travel. Carlquist (1966a, b) has also pointed out that mechanisms of dispersal tend to reduce or disappear in island species, but this would happen after inter-island dispersal and the patterns of adaptive radiation have been established.

Successfully radiated groups have obviously evolved quite rapidly, and short generation times would facilitate more rapid transmission of mutations and increase recombination, both of which could yield more rapid morphological/ecological adaptations. Annual plants would be more favorable in this regard, but the annual habit is very rare in oceanic islands. The greater occurrence of perennials ostensibly results from their ability to survive until favorable circumstances allow them to proliferate, reproduce, and disperse to new habitats, whereas annuals are dependent on seed set in each generation (assuming they do not produce a long-lived seed bank). Nearly all endemic species in the Juan Fernández Islands are perennials (Marticorena et al. 1998), the herbaceous perennial habit being mostly typical of radiated groups, with some exceptions. There are many examples of woody island groups being derived from herbaceous progenitors (Carlquist 1974), but the wood is often elaborated vascular and support tissue reflecting herbaceous origin. In *Dendroseris* (Asteraceae), for example, which is in the same tribe as the common herbaceous dandelion (*Taraxacum*), all species are woody but in the form of rosette trees with soft tissues (Carlquist 1967).

Breeding systems are also important to consider, especially mixed-mating systems (Goodwillie et al. 2005). One breeding system that would be advantageous for a colonizer is self-compatibility (SC) with mechanisms promoting outcrossing while being able to set some seed by selfing. While a strictly SC and highly selfing colonizer could establish a sexual population from even a single colonizer because it originated from a highly selfing source population, it would presumably have low levels of genome-wide diversity, and this could limit radiation, diversification, and speciation. A second preferred breeding system would be leaky self-incompatibility (so called pseudo-self-compatibility, PSC) (Crawford et al. 2009, 2011). Obligatory self-incompatible (SI) colonizers would be at a significant disadvantage because of the need to have at least two genetically cross-compatible individuals for a successful cross, which would be unlikely with chance dispersal to isolated archipelagos. PSC would be the preferred condition because it could result in some selfed progeny to help build up an immigrant population but would also normally restrict successful crosses to genetically distinct individuals. This would encourage genetic diversity, so important for eventual divergence.

Need for specialized pollinators would be a distinct disadvantage in an immigrant population. If a species has a very specific pollinator in the progenitor of the continental source area, it is almost certain that this pollinator will not be present in the island habitat. A generalized pollination system, therefore, would be much more suitable for successful colonization. An instructive comparison in this regard is between Asteraceae and Orchidaceae. The former have generalist pollination strategies and have been very successful in isolated oceanic islands, whereas the latter are dependent upon very specific pollinators and usually quite scarce in oceanic archipelagos, despite that the very small seeds of orchids should be easily dispersed by wind. Lack of required mycorrhizal fungi in the new environment may also be a limiting factor.

In addition to biological features of the immigrant plants themselves, the phylogenetic background of the progenitors is also important. One important consideration in this respect is that the progenitor stock should have a tendency for ecological differentiation. A lineage that has never diverged into ecologically distinct subgroups would be unlikely to adaptively radiate in islands no matter what the ecological opportunity. Another important consideration is that the progenitor lineage should have revealed a tendency for eco-geographic speciation. Groups that have relied on cycles of chromosomal change for speciation, such as with polyploidy or dysploidy, would not likely be successful in the island setting where such speciation is virtually non-existent, apparently under negative selection (Carr 1998; Stuessy and Crawford 1998). The point here is that eco-geographic isolation should typically result in morphological and genetic changes, as these are the primary modes of divergence via adaptive radiation. These aspects of continental progenitors have not previously been emphasized in explaining reasons for adaptive evolution in oceanic islands, but we believe them to be significant. This obviously requires having considerable phylogenetic knowledge about what the continental progenitors might be and their biological and ecological characteristics.

### Features of adaptively radiated groups in the Juan Fernández endemic flora

**Fig. 2:**
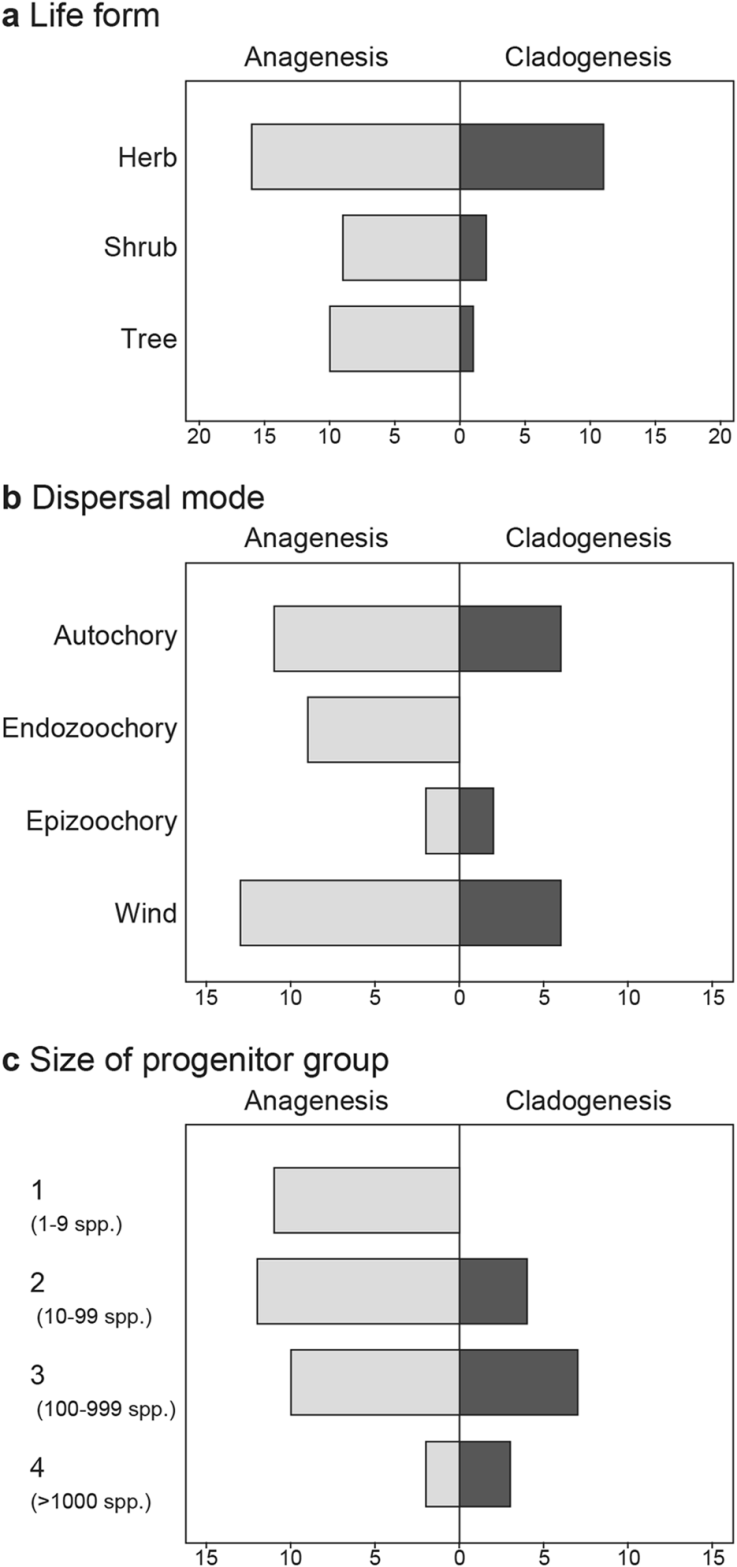
Correlations of biological and progenitor features with numbers of genera giving rise to anagenetically and cladogenetically derived endemic angiosperm species of the Juan Fernández Archipelago. **a** With life forms; **b** with mode of dispersal; **c** with size of progenitor group (1 = 1–9 species; 2 = 10–99; 3 = 100–999; 4 = > 1000)

We can examine the biological characteristics (Tables 1, 2) of the anagenetically derived and adaptively radiated (cladogenetic) endemic species of the Juan Fernández flora for clues to attributes involved with their modes of speciation. It is immediately obvious from the GGraph frequency plot (Fig. 2a) and a significant correlation (rho = 0.305, *p* < 0.05) between life form and mode of speciation that species involved with adaptive radiation are herbs rather than shrubs or trees. This supports the concept that rapid generation time is an important factor in stimulating accumulation of genetic change to permit adaptation to new environments. This is a conspicuous difference between species having evolved via the two processes.

The mode of dispersal of species in the islands may have affected patterns of gene flow and differentiation. It is likely that wind-dispersal and autochory have been important in the island settings as is apparently the case in the Juan Fernández Islands (see Bernardello et al. 2006). In this regard, we have treated all cases of mixed dispersal mode (anemo-/endozoochorous) as wind-dispersed. There are no entirely endozoochorous taxa among the cladogenetically derived species. There is, however, a clear association between endozoochory and the woody life forms (trees and shrubs) that are rare in the progenitors of cladogenetically derived species (Table 2). Apart from the high proportion of endozoochory in the anagenetically derived species, the GGraph frequency plot (Fig. 2b) shows a nearly bimodal distribution in both groups. Overall there is no correlation between dispersal mode and mode of speciation.

As for reproductive systems, little seems to differ between species originating cladogenetically or anagenetically. Although all endemic species of the flora have not been investigated for reproductive features, a good survey (in 39 genera) has been completed by Bernadello et al. (2001) for a number of aspects. The majority of species are self-compatible, although infrequently selfing. That is, pollen must be moved from the anther to the stigma of the same flower or from one flower to another within the same plant (geitonogamy). It is also notable that the three largest lineages in the archipelago, namely *Dendroseris, Erigeron* and *Robinsonia*, very likely originated from SI or PSC colonizers (Crawford et al. 2009). Self-compatibility has apparently evolved in one species of *Dendroseris* (Anderson et al. 2001). *Robinsonia*, being dioecious, is outcrossing, but the breeding system of *Erigeron* has not been studied. Nothing more is known about details of the genetic systems, but some sort of selfing mechanism seems typical for species of the flora, regardless of the type of speciation that occurred after arrival. For pollination, most of the endemic species of the flora are now wind-pollinated, although this may not be obvious from their morphology alone, which suggests ancestral insect pollination. Because of the virtual absence of insect pollinators in the archipelago, wind and bird pollination have become the rule. The more specific type of pollinator is hummingbirds (two species), but this pollinator shift from ancestral insect to island bird pollination has occurred about equally in anagenetically or cladogenetically derived species (Bernardello et al. 2001).

In addition to the features of successfully radiated groups in the Juan Fernández Archipelago, it is profitable to examine characteristics of their progenitors. The three genera with the largest adaptively radiated complexes in the endemic flora are *Dendroseris, Erigeron*, and *Robinsonia*. In a narrow sense of adaptive radiation, which requires larger complexes of divergent species (Losos and Mahler 2010), these would be the only genera in the archipelago that would represent this pattern. Six species of *Erigeron* are endemic in the Juan Fernández Islands, but they do not form an endemic genus. These species tie to other species of *Erigeron* (or perhaps even to *Conyza*; Andrus et al. 2009) in continental South America (Noyes 2000; Solbrig 1962). The available data suggest that the island complex derived from a single introduction (Noyes 2000). There are about 400 species in the genus with 27 species of *Erigeron* in the South American continent (Solbrig 1962), and these have colonized many different types of habitats, such as low marshy areas and wet slopes, coastal dunes, high elevation mountain habitats, and the lake region of southern Chile (Solbrig 1962). The species show different growth forms, from strictly herbaceous and annual (*E. leptorhizon*) to somewhat woody as cushion plants (*E. rosulatus*), subshrubs (e.g., *E. othonnaefolius*) or shrubs (e.g., *E. fasciculatus, E. luxurians*). The genus has clearly been evolutionarily successful in South America, with the *E. andicola* complex of closely related species inhabiting the southern Andes from central Chile and Argentina down into Patagonia. *Erigeron* has also colonized and speciated anagenetically in the Islas Malvinas (Falkland Islands) and the Galápagos Islands, indicating a strong ability for long distance dispersal. This all emphasizes that following dispersal to the Juan Fernández Islands, most probably to the younger island (Stuessy et al. 2017b); one of the exceptions to the “progression rule”, Funk and Wagner (1995), continued evolution via adaptive radiation took place.

The progenitors of *Dendroseris* have been investigated in some detail by Kim et al. (1996a, b, 2007), and the results suggest that the genus has evolved from *Sonchus* s.l., another large genus of some 100 species. The tie is sufficiently close, in fact, that Mejias and Kim (2012) have formally transferred the genus into *Sonchus*. The data do not show definitively from which part of *Sonchus* the species of *Dendroseris* may have come, but there is a strong possibility of derivation from the western Pacific from progenitors similar to the close generic relatives *Actites, Embergeria*, or *Kirkianella* (Kim et al. 1999a, b). In any event, within *Sonchus* s.l. have evolved many different forms in continental regions and in other island archipelagos, particularly the Canary Islands (*Dendrosonchus*; Aldridge 1978), which suggests that there would be no barrier to continued radiation when arriving successfully to Robinson Crusoe Island. It should be mentioned that species of *Dendrosonchus* have speciated at the diploid level whereas those within *Dendroseris* are all polyploids (Kim et al. 1999a, b; Stuessy et al. 2017b).

Progenitors of *Robinsonia* have been shown to be from the massive genus *Senecio* (Senecioneae), one of the largest genera of flowering plants with more than 1000 species and some 200 species in Chile alone (Cabrera 1949). Pelser et al. (2007, 2010a) have shown that *Robinsonia* has been derived from out of *Senecio*, but due to a limited sample size in the continent, it is impossible at this point to know from what part of the genus it has come. Because of being nested within *Senecio* and the desire to preserve strict holophyly, Pelser et al. (2010b) have elected to transfer all species of *Robinsonia* into *Senecio*, a taxonomic perspective with which we do not agree (Stuessy et al. 2017b). The important point is that *Senecio* has already adaptively radiated in many parts of the world, and especially in South America. This is a genus that has been enormously successful due to its ability to disperse, adapt to divergent habitats, and speciate rapidly. It comes as no surprise that such a lineage, once arriving first to the Robinson Crusoe Island, would speciate cladogenetically and adapt to different ecological zones when this island was younger. As mentioned earlier, at the present time, these broad areas have been greatly reduced over time, such that the species of *Robinsonia* are crowded in and around the main ridges of the island.

Conversely, it is profitable to examine the progenitors (close continental relatives) of endemic species in the Juan Fernández Islands that have speciated anagenetically, that is, that have not undergone adaptive radiation in the archipelago. Table 1 lists these endemics with their known habit, plus numbers of species in the continental source group. Two points are seen from these data. First, although some of the species are herbaceous, many are woody, either shrubs or trees. One genus is a palm, and another is a bamboo; if we exclude these two genera, then 15 (47%) are herbaceous, 14 (44%) either shrubs or trees, and 3 (9%) are mixed. This contrasts with the habit assessment of ancestral genera from which cladogenetic species have originated, whereby 12 (80%) are herbaceous, 1 (7%) contains shrubs and trees, and 2 (13%) are mixed. There is a clear trend, therefore, for adaptive radiation to take place within lineages that are herbaceous rather than woody, which surely relates to generation time and adaptability to differing ecological conditions. Second, the cladogenetically derived species come from more speciose progenitor groups, with an average of 449 species per genus (Table 2). In contrast, those anagenetically evolved species originate from genera with fewer species on average, here 180 (Table 1). Although there are clear exceptions, such as in *Ranunculus* with 600 species world-wide but only one endemic on Alejandro Selkirk Island, in general the anagenetically derived species do come from smaller, or less evolutionary successful, complexes. This is confirmed by the GGraph frequency plot (Fig. 2c) and a significant correlation (rho = 0.405, *p* < 0.01) between the progenitor group size and the mode of speciation. It is also the case that the larger number of anagenetic species occur on the older island, which might be expected. If anagenesis relies on dispersal, establishment, and change over time, then more opportunity has been provided on the older island for this type of process to occur.

Finally, we have investigated the performance of the three explanatory variables life form, dispersal mode, and progenitor group size in a generalized linear model (GLM). The fitted GLM was supported by an Omnibus test (Likelihood Ratio Chi-Square = 11.756, *df* = 3, *p* < 0.01). The estimated parameters are shown in Table 3. The progenitor group size turned out to be the only significant explaining variable (*p* < 0.05) in the GLM, whereas the performances of life form and dispersal mode were non-significant.

**Table 3:** Parameter estimates for the GLM using life form, dispersal mode, and the total number of species in progenitor genus (group) as explanatory variables

Parameter	Coefficient	Std. error	95% Wald confidence interval		Hypothesis test		
			Lower	Upper	Wald Chi square	*df*	*p* value
(Intercept)	6.413	2.2579	1.988	10.839	8.068	1	0.005**
Life form	− 0.816	0.5356	− 1.866	0.233	2.324	1	0.127
Dispersal mode	− 0.262	0.2913	− 0.833	0.309	0.807	1	0.369
Total number of species in progenitor genus (group)	− 1.120	0.4774	− 2.055	–0.184	5.499	1	0.019*

Response variable: mode of speciation

*Significant at the 0.05 level

**Significant at the 0.01 level

### Features of adaptively radiated groups in other oceanic archipelagos

The three island archipelagos that contain most of the classic examples of dramatic adaptive radiation are the Hawaiian Islands, Galápagos Islands, and the Canary Islands. These are certainly not the only island systems in which adaptive radiation has occurred, but they do contain numerous examples of this phenomenon for comparison.

Perhaps the most spectacular plant radiation in oceanic islands is the lobelioid complex of the Hawaiian archipelago, consisting of six genera and 126 species, which has been suggested to have derived from a single immigration (Givnish et al. 2009). The life forms that have evolved in this group include trees, treelets, shrubs, and even a few epiphytes and vines. They inhabit alpine bogs, seacliffs, and essentially all manner of moist environments within the archipelago. That this massive adaptively radiated complex could evolve within approximately 13 million years (Givnish et al. 2009) from a single introduction only emphasizes the potential of morphological change under intense directional selection into distinct habitats. Significant, however, is that the lineage was already pre-adapted to this type of speciation. There is little doubt that the Hawaiian assemblage has evolved from *Lobelia*, and three geographic source areas are most likely: Africa, the Pacific, or southern Asia (Givnish et al. 2009). Among the c. 300 species of the genus, numerous morphological and ecological variants exist, including the conspicuous giant lobelias of Africa (Antonelli 2009). Once a founding taxon from this successful lineage arrived to the diverse habitats offered in the Hawaiian archipelago, an explosive radiation occurred.

In the Canary Islands, a well-studied plant group is *Echium* (Boraginaceae). This genus has 27 endemic species in the Macaronesian archipelagos (Bramwell 1972), and there are c. 30 species found in the circum-Mediterranean-west Asian regions (Böhle et al. 1996). The genus has been successful in continental areas with the habit of species ranging from creeping to spreading or even upright. In the Canary Islands, endemic species of *Echium* have evolved into larger forms, with some branching, and others with a large central woody axis with massive inflorescences. The continental species of the genus have been reported as preferentially inbreeding (Böhle et al. 1996), whereas those in the islands are outcrossing, but with 0–11% fertile seed in selfings. This might suggest that the island endemics could possibly be PSC. In any event, the progenitor lineage of the island species is a very successful one, occurring in different habitats and representing a diverse collection of different growth forms.

For the Galápagos Islands, the most conspicuous plant radiation has occurred in the genus *Scalesia* (Asteraceae) with approximately 15 endemic species (Eliasson 1974; Itow 1995). Species are either trees (three species) restricted to mid-elevation moist habitats, or shrubs (twelve species) occurring in dry woodlands. Schilling et al. (1994) carried out cpDNA restriction site analyses of species of *Scalesia* and presumptive continental relatives, and the genus *Pappobolus* was suggested to be closest. Blaschke and Sanders (2009) completed a morphological phylogenetic analysis of generic and specific relationships involving *Scalesia* and other genera of Helianthinae, and they suggested that *Scalesia* might be close or closer to *Simsia* or species of *Viquiera*. We regard these results to be less phylogenetically informative than the cpDNA analyses, but even if it were the case that *Scalesia* derived from either of these other genera, they, too, are large and diverse. *Pappobolus* has 38 species from Ecuador, Peru and Bolivia (Panero 1992), and more importantly, it has radiated throughout the Andean chain. Species occur from 650 to 4200 m and are found in many different habitats from xerophytic forest to moist jalca, and these include desert scrub, thorn woodland, steppe, and subalpine moist forest. It is not at all difficult to imagine a propagule dispersing from this Andean area to the Galápagos Islands and adaptively radiating among the diverse habitats available in the archipelago. Chamorro et al. (2012) cite self-compatibility and autonomous self-pollination in *S. affinis, S. aspera, S. bauri, S. cordata, S. helleri*, and *S. pedunculata. Pappobolus hypargyreus* has been determined to be self-compatible (Heiser 1957), but Panero (1992) reports likely self-incompatibility in *P. hutchisonii* and *P. juncosae*. Male sterility of some florets in capitula (rendering these heads gynomonoecious and hence promoting geitonogamy and outcrossing) of some species has also been described (Panero 1992).

## Ecological context for island endemics that have originated by adaptive radiation

**Fig. 3:**
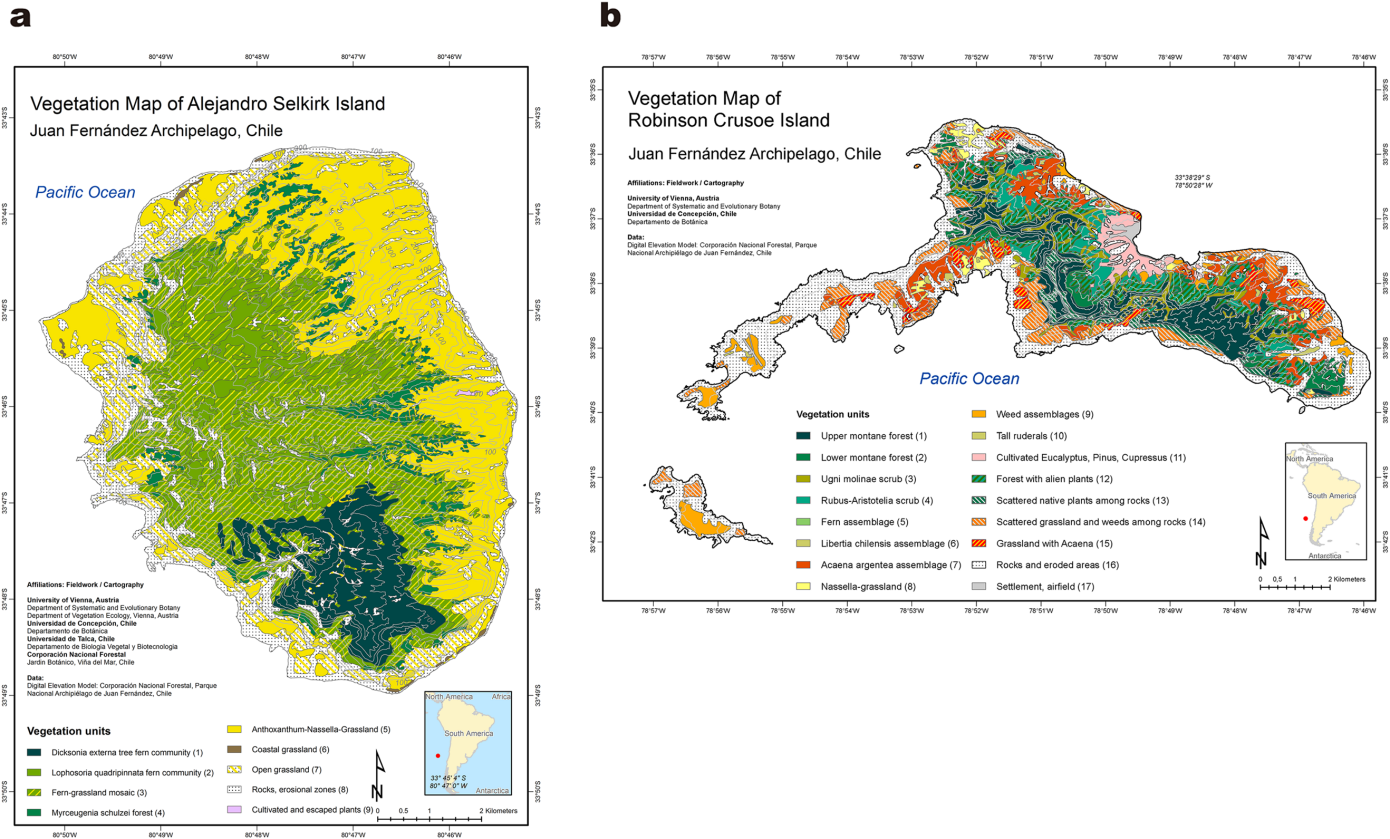
Vegetation maps of **a** Alejandro Selkirk Island (Greimler et al. 2013) and **b** Robinson Crusoe Island (modified from Greimler et al. 2002)

Of fundamental importance for adaptive radiation is that newly derived species diverge into distinct habitats. This is, in fact, one of the key aspects of adaptive radiation. The classical examples of island plant radiation discussed above all show that species have become adapted to diverse environments. In the Juan Fernández Islands, the most suitable approach is to examine the habitat partitioning that now exists (using recent vegetation analyses and maps, Fig. 3; Greimler et al. 2002, 2013) and to determine how cladogenetically derived species correlate with these zones. Of particular interest is a comparison of the three largest genera of the archipelago. *Dendroseris* and *Robinsonia* have adaptively radiated on the older island (Robinson Crusoe) and *Erigeron* has done so on Alejandro Selkirk, the younger island. All the genera are in Asteraceae, but in different tribes of the family (Cichorieae, Senecioneae, Astereae, respectively).

The most instructive case is *Erigeron* on the younger island, Alejandro Selkirk. Notice that the vegetation map (Fig. 3a) reveals clearly demarcated habitats in reasonably large sections on the island. This is what might be expected for a young island (c. 1 million years old) in which vegetation has developed, but where erosion and subsidence have not yet begun to dramatically modify the original patterns of vegetation. From the map, species of *Erigeron* have diverged into major different zones: *E. rupicola* along the coast (cliffs and open grassland); *E. stuessyi* in the inner parts of deep ravines (quebradas), shown as the *Lophosoria quadripinnata* fern community and the *Myrceugenia schulzei* forest; *E. fernandezia* on steep walls of the canyons together with fragments of the *Anthoxanthum*-*Nasella*-grassland embedded essentially in the *Myrceugenia schulzei* forest; and the *E. ingae* complex (*E. ingae, E. luteoviridis, E. turricola*) in the fern-grassland mosaic at higher elevations. The only major zone into which *Erigeron* has apparently not radiated is the *Dicksonia externa* tree fern community, which is a dense habitat that may not offer enough light for *Erigeron* to survive.

The situation with *Dendroseris* and *Robinsonia* is quite different. These are endemic genera that contain 11 and 8 species, respectively, with 8 and 7 species that have adaptively radiated on Robinson Crusoe Island. The other four species occur on Alejandro Selkirk Island and all (*D. gigantea, D. macrophylla, D. regia, R. masafuerae*) have originated anagenetically from progenitors on Robinson Crusoe Island. Associated species and soil mineral studies have shown (Sanders et al. 1987) that species in these two genera on Robinson Crusoe Island, despite considerable morphological differences among them, do not at this time show dramatic differences among the environments in which they are found. In *Dendroseris, D. litoralis* is confined to the open shoreline, *D. pruinata* grows along coastal cliffs, and *D. marginata* favors rocky outcrops within forested areas, but the other species are found scattered within the native forest. Part of the problem is that a number of these species are critically endangered, and often occur only individually, which makes deciphering of population tendencies difficult. As for *Robinsoni*a, Carlquist (1974) noticed different anatomical features in leaves of species, which he attempted to correlate with different habitat tendencies reported by Skottsberg (1921) and other previous collectors (he, himself, never visited the archipelago). What we have observed in our expeditions to the island is that most species of *Robinsonia* occur within the native forest, but that *R. gayana* occurs on exposed basaltic ridges and *R. gracilis* in open forested ridge areas above 700 m.

The problem is that at the present time (after 4 million years) on Robinson Crusoe Island, there are no longer large and distinct vegetation regions similar to those seen on the younger island (Fig. 3a, b). In fact, all of the native flora is confined to the narrow bank of original forest running NE–SW along the remaining central ridges (Fig. 3b). This pattern, however, is what one might anticipate with a consideration of the geological history of the islands. The younger island has undergone only a small loss of surface area during its 1–2 million years of existence, whereas the older island may have suffered much more (as much as 95%; Stuessy et al. 1998). One speculates that due to so much environmental alteration, the flora has been reduced in area (Stuessy 2007). Species have relinquished their original geographic distributions, surviving now more or less in a refugium. Just looking at Fig. 3b gives the impression that the vegetation has been pushed together around the remaining higher ridges of the island, providing a dramatic contrast with the patterns on the younger island (Fig. 3a).

## Summary of factors that are favorable for adaptive radiation in oceanic archipelagos

Examination of the factors that are associated with adaptively radiated species complexes in the Juan Fernández Islands allows development of a profile that drives this type of speciation not only in this archipelago but also in any oceanic island. The general factors obviously relate to island environment and biological characteristics of immigrant populations.

A fundamental aspect of adaptive radiation is that varied environments must be present in the island setting. The most conspicuous cases of adaptive radiation in plants of oceanic islands are in the Hawaiian Islands, Galápagos Island, and the Canary Islands. The environmental heterogeneity in all three archipelagoes is striking, and this correlates with high levels of endemism and cladogenetic origins of the flora. Furthermore, these islands have undergone recent volcanic activity, which provides for newly formed habitats and an increasing number of ecological opportunities.

Another significant aspect of adaptive radiation is the series of biological attributes of the colonizing lineages. Most successful colonists are perennials, which provides a mechanism for establishment and continued survival both within the initial environment and also in derivative habitats after dispersal within and between islands. These species are herbaceous, or have become woody during diversification, but always retaining rapid generation turnover. It is very unusual to have an adaptively radiated complex in true woody lineages, and no large trees have radiated in the Juan Fernández Islands. Also advantageous for immigrant populations to be able to set seed from selfing while at the same time having sufficient genome-wide heterozygosity to facilitate divergence and speciation. One mechanism is SC with capacity to both outcross and self, and another is PSC. Both provide flexible genetic systems that promote outcrossing while at the same time allowing for occasional selfing. Difficult to define and quantify are the physiological tolerances, so important for allowing adaptations to different environments. An immigrant lineage that has only narrow physiological tolerances will likely not adapt well to different environments, even if they successfully disperse into them. Such flexibility would extend to water relations, which would have an anatomical basis in the vascular system (e.g., Carlquist 1975). An additional advantage would be conferred on lineages that showed tendencies for modifications of structural change, such as in branching, vining, production of long stems (as in rosette trees), etc. Tolerance of different mineral regimes in the substrate would also be important, but most significant would be variation that allows for photosynthesis under different light and humidity conditions (Santiago and Kim 2009). Plants also have a high capacity for plasticity (Schlichting and Pigliucci 1998; Schlichting and Wund 2014), which adds to the spectrum of potential tolerances in different habitats. Finally, variation in production of secondary plant metabolites, which are so diverse in vascular plants, would allow defense and metabolic functions to be more flexible and efficacious in new environments. Changes have doubtless occurred in phytochemical profiles in colonizing lineages in the absence of the original spectrum of continental herbivores and pests (insects, viruses, bacteria, fungi, herbivores), but a rich cocktail of compounds still prevails (e.g., Bohm 1998).

The ability to successfully radiate within a new island setting is obviously not a simple matter, and many factors are involved, such as mentioned above. Because of this complexity, it is not likely that repeating cycles of adaptive radiation would take place within the same island, or between islands of the same archipelago. That is, once a group radiates successfully within an island, even though propagules successfully disperse from these newly evolved species to another island, which may also contain ecological opportunity, it is rare that another cycle of adaptive radiation ensues. Such a phenomenon, called the “taxon cycle” by Wilson (1961), seems generally uncommon and does not exist in the Juan Fernández Archipelago. Quite a number of examples exist of dispersal from Robinson Crusoe Island to Alejandro Selkirk Island, such as in *Robinsonia* and *Dendroseris*, but the only divergence that occurs has been anagenetic. Part of the reason for this limitation in cycles of adaptive radiation may relate to the young age of Alejandro Selkirk Island, but there does seem to be an “exhaustion” of genetic potential in the first radiation that makes subsequent radiations unlikely. Each of the radiated species on the older island has already gone through a founder effect and has been channeled via stringent directional selection, and with further dispersal to the younger island, another founder effect occurs. These genetic restrictions may be at least part of the reason for the complete lack of cycles of adaptive radiation in the archipelago.

In conclusion, the genera that have adaptively radiated in the Juan Fernández Islands are those that are in families that have adaptively radiated elsewhere in the world. That is, in the islands they are simply continuing their mode of speciation exhibited elsewhere, but on a different geographic scale. One would not expect an immigrant from a very successful ecologically diverse genus, newly arriving on a new oceanic island containing diverse habitats, to remain in only one ecological zone for very long. What differs is that ecological zones in islands can be small and located very near each other, such that dispersal into different zones is facilitated and change can occur rapidly due to short generation times and intense directional selection. Adaptive radiation can occur in continental regions, too, such as in the environmentally diverse Andes of South America (e.g., in *Lupinus*; Hughes and Eastwood 2006), or even on a continental scale in South America (e.g., in *Hypochaeris*; Urtubey et al. 2018). The bottom line is that adaptive radiation occurs when an already successful evolutionary lineage meets ecological opportunity, whether in oceanic islands or elsewhere on the planet. This is similar to the idea of “extrinsic conditions and intrinsic traits” suggested by Bouchenak-Khelladi et al. (2015) for explaining evolutionary radiations in a broader context.

### Note on statistics

The information given in Tables 1 and 2 was categorized in the following way: (1) the typical life form of the ancestral genus (group) was classified as tree, shrub (incl. subshrub, cane), and herb. Multiple states (e.g., herbs to trees) were coded as trees. (2) The total number of species in progenitor genus (group) was classified in an exponential way: 1 for 1–9 species, 2 for 10–99, 3 for 100–999, 4 for > 1000. (3) Dispersal mode of island taxa was coded by 1 for anemochory (including mixed modes with anemochory involved), 2 for epizoochory, 3 for endozoochory, and 4 for autochory. The variables were examined using the options Examine, Correlations (non-parametric Spearmans Rank Correlation, rho), and GGraph of IBM SPSS ver. 24. We used a generalized linear model (GLM) with binomial distribution of the response variable and a logistic regression (logit-link) to evaluate the effects of three categorical explanatory variables. All data on the explanatory variables are given in Tables 1 and 2. To gain confidence in our model a likelihood ratio Chi square (Omnibus) test was used to compare the full with a reduced model containing only an intercept term. All statistics were calculated with IBM SPSS ver. 24.
