# Correction to: Factors driving adaptive radiation in plants of oceanic islands: a case study from the Juan Fernández Archipelago

**DOI:** 10.1007/s10265-019-01092-z

**Published:** 2019-02-25

**Authors:** Koji Takayama, Daniel J. Crawford, Patricio López-Sepúlveda, Josef Greimler, Tod F. Stuessy

**Affiliations:** 1 Graduate School of Science, Kyoto University, Kitashirakawa Oiwake-cho, Sakyo-ku, Kyoto, 606-8502 Japan; 2 Department of Ecology and Evolutionary Biology, Biodiversity Institute, University of Kansas, Lawrence, KS 60045 USA; 3 Department of Botany, University of Concepción, Casilla 160-C, Concepción, Chile; 4 Department of Botany and Biodiversity Research, University of Vienna, Rennweg 14, 1030 Vienna, Austria; 5 Herbarium and Department of Evolution, Ecology, and Organismal Biology, The Ohio State University, 1315 Kinnear Road, Columbus, OH 43212 USA

## Correction to: Journal of Plant Research 10.1007/s10265-018-1023-z

The article Factors driving adaptive radiation in plants of oceanic islands: a case study from the Juan Fernández Archipelago, written by Koji Takayama, Daniel J. Crawford, Patricio López‑Sepúlveda, Josef Greimler, Tod F. Stuessy was originally published electronically on the publisher’s internet portal (currently SpringerLink) on 13 March 2018 without open access.

With the author(s)’ decision to opt for Open Choice the copyright of the article changed on 25 February 2019 to © The Author(s) [Year] and the article is forthwith distributed under the terms of the Creative Commons Attribution 4.0 International License (http://creativecommons.org/licenses/by/4.0/), which permits use, duplication, adaptation, distribution and reproduction in any medium or format, as long as you give appropriate credit to the original author(s) and the source, provide a link to the Creative Commons license and indicate if changes were made.

The original article has been corrected.

